# A novel loss-of-function mutation of the voltage-gated potassium channel Kv10.2 involved in epilepsy and autism

**DOI:** 10.1186/s13023-022-02499-z

**Published:** 2022-09-06

**Authors:** Jesús Galán-Vidal, Paula G. Socuéllamos, María Baena-Nuevo, Lizbeth Contreras, Teresa González, María S. Pérez-Poyato, Carmen Valenzuela, Domingo González-Lamuño, Alberto Gandarillas

**Affiliations:** 1 Cell Cycle, Stem Cell Fate and Cancer Laboratory, Institute for Research Marqués de Valdecilla (IDIVAL), 39011 Santander, Spain; 2 Instituto de Investigaciones Biomédicas Alberto Sols, IIBM, CSIC-UAM, Madrid, Spain; 3 Spanish Network for Biomedical Research in Cardiovascular Research (CIBERCV), Instituto de Salud Carlos III, Madrid, Spain; 4 Neuropediatric, University Hospital Marqués de Valdecilla, 39008 Santander, Spain; 5 Paediatric Department, University of Cantabria University, Marqués de Valdecilla Hospital, 39008 Santander, Spain; 6 INSERM, Occitanie Méditerranée, 34394 Montpellier, France

## Abstract

**Background:**

Novel developmental mutations associated with disease are a continuous challenge in medicine. Clinical consequences caused by these mutations include neuron and cognitive alterations that can lead to epilepsy or autism spectrum disorders. Often, it is difficult to identify the physiological defects and the appropriate treatments.

**Results:**

We have isolated and cultured primary cells from the skin of a patient with combined epilepsy and autism syndrome. A mutation in the potassium channel protein Kv10.2 was identified. We have characterised the alteration of the mutant channel and found that it causes loss of function (LOF). Primary cells from the skin displayed a very striking growth defect and increased differentiation. In vitro treatment with various carbonic anhydrase inhibitors with various degrees of specificity for potassium channels, (Brinzolamide, Acetazolamide, Retigabine) restored the activation capacity of the mutated channel. Interestingly, the drugs also recovered in vitro the expansion capacity of the mutated skin cells. Furthermore, treatment with Acetazolamide clearly improved the patient regarding epilepsy and cognitive skills. When the treatment was temporarily halted the syndrome worsened again.

**Conclusions:**

By in vitro studying primary cells from the patient and the activation capacity of the mutated protein, we could first, find a readout for the cellular defects and second, test pharmaceutical treatments that proved to be beneficial. The results show the involvement of a novel LOF mutation of a Potassium channel in autism syndrome with epilepsy and the great potential of in vitro cultures of primary cells in personalised medicine of rare diseases.

## Introduction

Autism spectrum disorders (ASD) affect normal brain development. ASD is characterised by deficits in social interaction, delayed communication skills and repetitive patterns of behaviour [1, 2]. Epilepsy encompasses a group of neurological disorders characterised by recurrent epileptic seizures. These seizures cause abnormal neuroelectric flows, resulting in physical injuries and neuronal disabilities [3]. The co-existence of epilepsy and ASD has long been recognised, frequently presented with other developmental disabilities such as developmental delay or intellectual disability [4, 5]. However, the molecular mechanisms involved in the combined development of epilepsy and ASD are unclear. The identification of associated genetic mutations plays a key role in the understanding, diagnosis and treatment of these disorders.

The term channelopathy refers to a spectrum of disorders caused by dysfunction of ion channels [6]. Ion channels are transmembrane proteins that facilitate a specific inflow/outflow of ions according to their electrochemical gradient. Genetic brain channelopathies arise from inherited or de novo mutations of ion channel genes within the central nervous system. These mutations elicit homeostatic imbalances in membrane excitability, commonly related with epilepsy, ASDs, intellectual disability or ataxia. The role of potassium channels has been highlighted within the described channelopathies, because of their function in the recovery of the resting membrane potential [7–9].

The Kv10.2 channel (also known as Eag2, encoded by the *KNCH5* gene) belongs to the ether-à-go-go (Eag) potassium channel family, within the superfamily of voltage-gated potassium (Kv) channels. Eag channel family consists of two known members in human, Kv10.1 and Kv10.2 [10, 11]. Kv10.2 is widely expressed in the central nervous system and organs such as skeletal muscle, heart and lung, playing a key role in setting membrane potentials and action potential repolarisation [12]. Kv10.2 has been reported to participate in tumour growth, cell size control and mitotic entry in medulloblastoma and has been proposed as a tumour marker and a therapeutic target [13–15]. However, its physiological function and role in neurological disorders is not fully understood.

A mutation in Kv10.2 has only been reported once before in human, a Kv10.2 gain of function (GOF) mutation was identified in a child with epileptic encephalopathy [16, 17]. Here we report a case of a child that presented at the age of two years old epilepsy and mild developmental delay. Although epilepsy was under apparent good clinical control, the child displayed language and motor regression, ataxia and a progressive autistic behaviour. A genetic study identified a mutation in the *KCNH5* gene, resulting in a punctual substitution in the Kv10.2 channel. By using cDNA constructs of the wild type or the mutated protein, we performed electrophysiological analyses *in cellula*. This demonstrated a loss of function (LOF) of the mutant channel. In addition, we isolated and cultured cells from the patient displaying significant growth and differentiation defects. Proliferation was not seemingly affected therefore we hypothesise that the channel dysfunction might affect the shape and spreading of cells via the cytoskeleton. Interestingly, pharmacological chemicals that promote potassium intake recovered growth expansion proprieties of the skin cells and also the activation capacity of mutant Kv10.2. Following the in vitro observations, the patient was treated with one such drugs, what achieved significant improvement. Therefore, we show that pharmaceutical rescue of a Kv10.2 mutant channel improved the clinical evolution of an infant with an epilepsy-cognitive syndrome. We discuss the potential implications of the cellular defects observed into the molecular biology of rare genetic neurological disorders.

## Results

An 11-year-old boy patient displayed moderate to severe developmental delay, autism, epilepsy and minimal physical dysmorphia and hyperlaxity during physical exploration. From 2 years of age onwards, after childhood episodic epilepsy attacks, he presented a severe chronic neuro-developmental complex disorder phenotype with intellectual disability and signs of the autism spectrum. The patient also displayed spinocerebellar ataxia, losses of motor skills and language regression until total loss at the age of 6. No structural alterations were observed during brain MRI (Magnetic Resonance Imaging; not shown). The karyotype test did not show any disturbance and biochemical and metabolic profiles were normal.

The patient received various antiepileptic treatments with variable impact (see Materials and methods). An interesting observation was the differential response to the same compound in different drug vehicles. While valproate (VPA) treatment of the commercial brand Depakine® (Sanofi S.A., France) was effective in controlling epilepsy, a generic VPA preparation increased the frequency of epileptic seizures and deteriorated neurodevelopmental parameters. The different response to a same active principle just depending on the drug vehicle was a determining hint to suspect about a possible channelopathy.

Next-generation sequencing (NGS), in particular Exome sequencing (ES), is a useful tool to identify genetic mutations that may cause neurologic diseases [18–20]. To identify candidate variants with possible functional significance, we performed two different ES analyses, one of them in a trio framework covering the unaffected parents. We found five interesting non-synonymous mutations: two compound heterozygous mutations with one allele inherited from each parent, and one heterozygous mutation inherited from the mother in a gene with a potential role in neurodevelopment disorders (Table 1). The frequency of the Calcium Voltage-Gated Channel Subunit *CACNA1I* A583T variant is relatively common (gnomAD: 0.002421; TOPMED: 0.003763), close to be considered as a polymorphism (frequency ≥ 0.01). Both variants in Glycogen Synthase Kinase 3 Beta-Interacting NIN gene are already registered in gnomAD and TOPMED database. The only variant not previously reported was the one in KCNH5 gene.

**Table 1:** Summary of candidate variants identified and in silico prediction scores in the patient

Gene	Protein ID	DNA change	AA change	gnomAD	TOPMED	GERP	CADD	REVEL
*CACNA1I*	Q9P0X4	82C>T	P28S	n.d	8.00E−06	1.27	14.69	0.350
		1747G>A	A583T	2.42E−03	3.76E−03	−0.55	0.67	0.244
*NIN*	Q8N4C6	5264C>T	A1755V	2.10E−05	1.10E−05	4.50	19.36	0.078
		6348A>G	I2116M	2.10E−05	1.10E−05	1.79	13.81	0.218
*KCNH5*	Q8NCM2	2566A>C	N856H	n.d	n.d	5.92	21.50	0.531

gnomAD and TOPMED databases gather previously reported variation frequencies. GERP tool display a conservation score for a nucleotide position, values above 3 are considered as highly conserved. CADD tool scores the predicted deleteriousness of a variant (SNP or indels), values above 20 can be considered as “*likely deleterious*”. REVEL method combines 13 individual tools to predict the pathogenicity of missense variants, with scores from 0 to 1, it is estimated “*likely disease causing*” when above 0.5

We performed in silico predictions to evaluate the deleteriousness of the observed mutations (GERP, CADD and REVEL; Table 1). Scores obtained for *NIN* and *CACNA1I* mutations did not seem to be deleterious. Furthermore, neither the biological role of NIN nor NIN variant phenotypes reported in ClinVar (219 results) seems to be related with the disease of the patient. With regard to *CACNA1I,* none of the 47 results gathered in ClinVar were related with epilepsy, just one result was found as ‘*Global developmental delay’*. Otherwise, all the prediction scores for KCNH5 variant displayed values above the threshold considered as deleterious. Furthermore, 368 of 395 *KCNH5* transcript variants found in ClinVar were reported as ‘*Early infantile epileptic encephalopathy with suppression bursts’*. Therefore, although the healthy mother contained this variant, we considered that KCNH5 mutation holds a role in the disease of the patient, possibly bolstered by another genetic alteration that we did not detect.

KCNH channels are included into the cyclic nucleotide-binding domain (CNBD) channels family [21]. However, KCNH channels do not bind cyclin nucleotides, although they have a cyclic nucleotide-binding homology domain (CNBHD) [22]. The C-terminal CNBD/CNBHD domain interacts with pore opening [23]. Also, the distal C-terminal portion of the CNBHD forms a coiled-coil domain which seems to be necessary for channel tetramerisation. In analogy with the structure of Kv10.1 channel, the Kv10.2 N856H mutation, located closely to the intracellular C-terminal domain, might be part of the CNBHD domain or the terminal coiled-coil domain.

With the above premises, we aimed to investigate whether the *KCNH5* mutation affected the function of the channel. We performed in vitro electrophysiological experiments to compare the properties of N856H Kv10.2 mutant channel to the wild type (WT) Kv10.2 one. To this end, we cloned the mutated form into a mammalian expressing vector. We then overexpressed this construct, or the wild type gene, in COS7 cells, as described in Material and Methods.

**Fig. 1:**
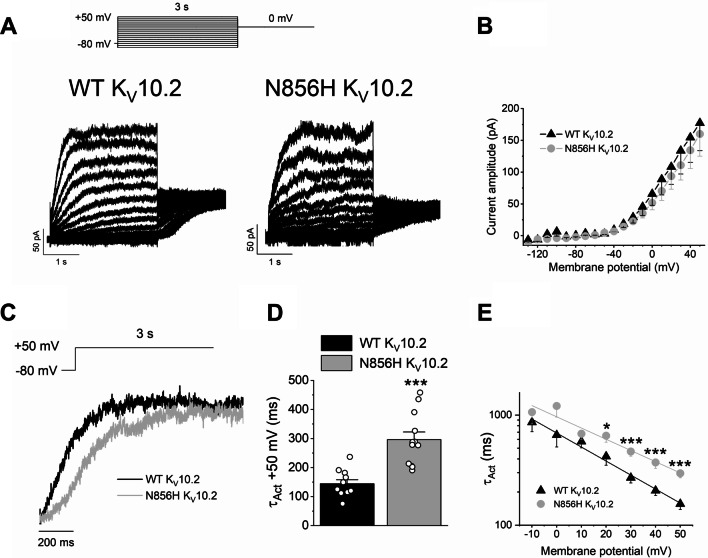
Electrophysiological properties of WT and N856H Kv10.2 channels expressed in COS7 cells. **A** Current recordings obtained in COS7 cells transfected with WT or N856H Kv10.2, as indicated, after applying the pulse protocol shown on the top. **B** Current–voltage relationship obtained after plotting the amplitude of the current measured at the end of the 3-s pulses versus membrane potential, as in **A**. **C** Normalised current recordings obtained after depolarising cells expressing WT or N856H from −80 mV to + 50 mV. **D** Time constant of activation of WT or N856H at + 50 mV. **E** Voltage dependence of the time constant of activation of WT or N856H. *n* = 12 for WT Kv10.2, *n* = 11 for N856H Kv10.2. *p < 0.05, **p < 0.01, ***p < 0.001

Voltage currents were elicited by 3-s pulses to potentials between −130 and + 50 mV from a holding potential of −80 mV in 10 mV steps, followed by a pulse to 0 mV. After applying this voltage pulse protocol, the slowly activating outward current generated by N856H Kv10.2 mutant channels resulted to be slower than in WT Kv10.2 channels (Fig. 1A). The current–voltage relationship (I-V) was obtained after plotting the amplitude of the current measured at the end of the 3-s pulses versus the membrane potential (Fig. 1B). The magnitude of the current resulted to be similar for both channels. However, the activation kinetics of the current generated by N856H Kv10.2 channels resulted significantly slower than that generated by the activation of WT channels (Fig. 1C, D). This effect was significant at membrane potentials positive to + 20 mV (p < 0.05) (Fig. 1E). These results show that the mutation causes a LOF of the channel.

We questioned whether the mutation of the Kv10.2 channel resulted in cellular defects. To this aim, we isolated cells from the epidermis of the skin of the patient and established primary cultures. We then studied the morphology and behaviour of the mutant keratinocytes. The epidermis is easily accessible and allows isolation and expansion of keratinocytes to establish an in vitro model of the disorder. Primary keratinocytes from the patient (Kmut) were compared with control keratinocytes from the skin of two healthy children with no alterations detected (KCT).

**Fig. 2:**
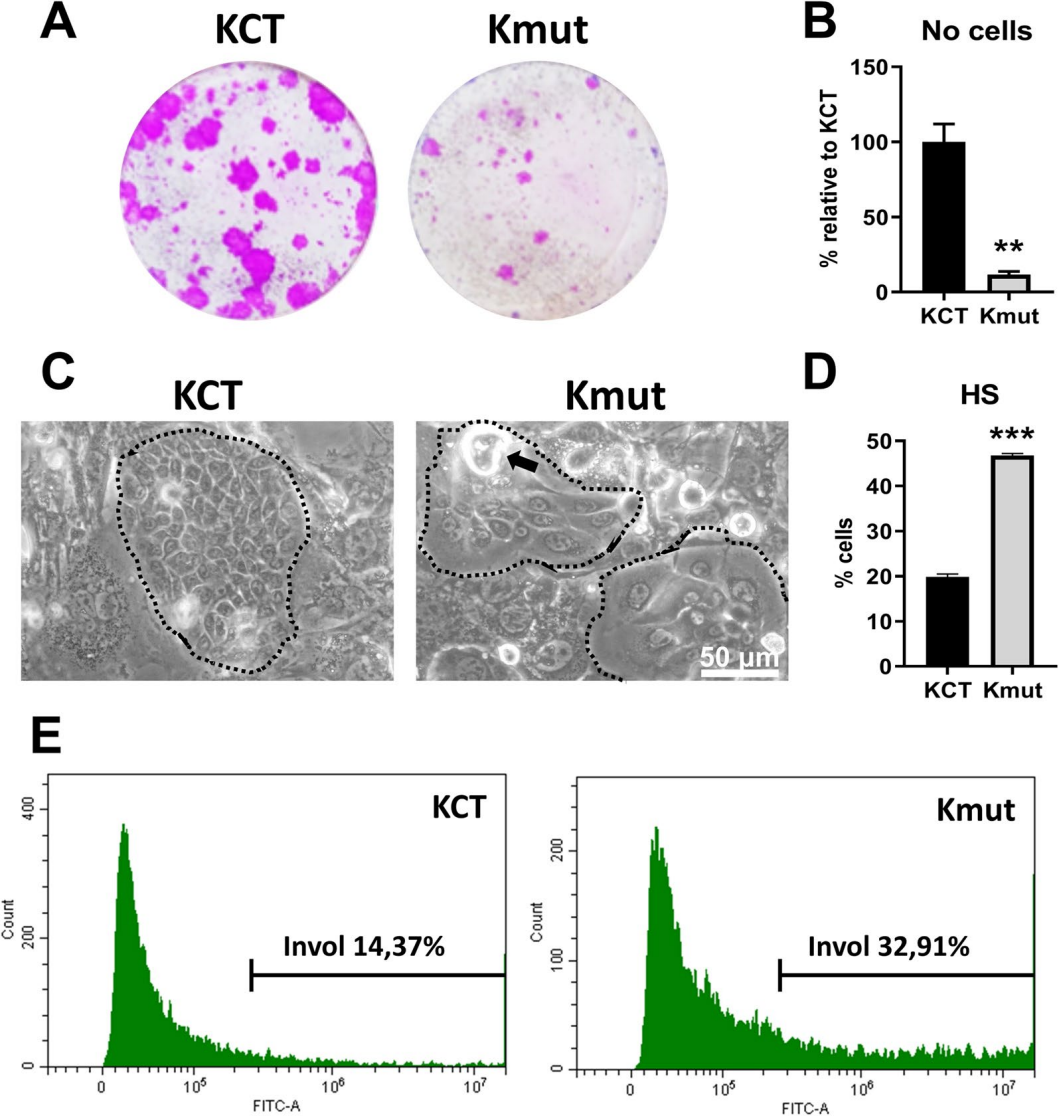
N856H Kv10.2 mutant primary cells isolated from the patient display a strong growth defect and enhanced terminal differentiation. Healthy keratinocytes from two healthy infantile donors were used as control (KCT) compared with mut keratinocytes containing the mutation (Kmut). **A** Representative clonal expansion, as monitored by clonogenicity assays (*n* = 3 triplicates per essay, 2 essays per healthy strain). **B** Number of cells harvested in **A** at confluence of healthy strains, relative to KCT (*n* = 2, 2 essays per healthy strain). **C** Representative phase contrast microphotograph of KCT or Kmut cells as indicated. Dotted lines enclose keratinocyte colonies, surrounded by a fibroblastic feeder layer (see “Materials and methods”). Black arrow points to a typical detaching differentiated cell that were very frequent in the mutant cultures. Scale bar, 50 μm. **D** Bar histogram shows the percentage of cells with high size and complexity, by light scattering parameters (HS), typical of differentiated keratinocytes, as quantified by flow-cytometry (*n* = 2, as in **A**). **E** Expression of the epidermoid differentiation marker involucrin n KCT or Kmut, as indicated, measured by immunodetection and flow cytometry (representative of 3 independent essays). **p < 0.01, ***p < 0.001

The growth capacity of Kmut was greatly reduced, as monitored by clonogenicity assays (clonal expansion; Fig. 2A) and by the number of growing cells harvested from the plate at the control confluence (Fig. 2B). The in vitro phenotype of primary keratinocytes in these conditions is a useful indicative of the proliferation/differentiation potential of the original tissue. While KCT cells grew tightly packed within large colonies, Kmut cells displayed an altered morphology growing in small colonies. During terminal squamous differentiation, keratinocytes lose their proliferative capacity, increase in size and stratify before turning into corneocytes and shed from the surface [24]. Kmut colonies were composed by large differentiating-like cells less tightly attached to each other (Fig. 2C). Kmut also cells stratified prematurely and more profusely (Fig. 2C; black arrow).

To monitor and quantitate the differences in cellular morphology and behaviour, cell size and complexity was analysed by flow cytometry and light scattering parameters. It is well established that keratinocytes become larger and more complex and acquire high light scatter proprieties (HS) as they differentiate [25]. As shown in Fig. 2D, the percentage of Kmut cells with HS values doubled the KCT reference measurements. Involucrin, a precursor protein of the cornified cell envelope, broadly used as a terminal squamous differentiation marker [25, 26], was also increased in Kmut compared to KCT (Fig. 2E). All measured parameters consistently demonstrated a growth defect and an enhanced index of terminal differentiation in the mutant cells.

Carbonic anhydrase inhibitors (CAIs) have been used in the clinic for a wide range of disorders. Among CAIs, sulphonamides are used as diuretics, antiglaucoma or antiepileptic [27]. Acetazolamide (AZA) is a sulphonamide approved as CAI by the Food and Drug Administration (FDA) for the treatment of epilepsy [28, 29]. The inhibition of carbonic anhydrase by AZA might change intracellular pH, with an effect over the transmembrane potential. Among AZA physiological effects, a drop in serum potassium level in the extracellular environment was described, suggesting an increase in the cellular potassium intake [30, 31]. Closely related to AZA, brinzolamide (BZA) is a sulphonamide drug used as CAI with potential use as antiepileptic [32, 33]. In an initial phase to search for a drug efficient in treating the patient for the Kv10.2 channel dysfunction, we went on treating primary skin keratinocytes of the patient with these drugs. In addition, we were interested in testing the retigabine (RTG) compound. This drug has proven to specifically open another closely related family of voltage-gated potassium channels, Kv7.2–5 [34–37], and allows higher dosed treatments. However, RTG is not authorised to treat minors.

**Fig. 3:**
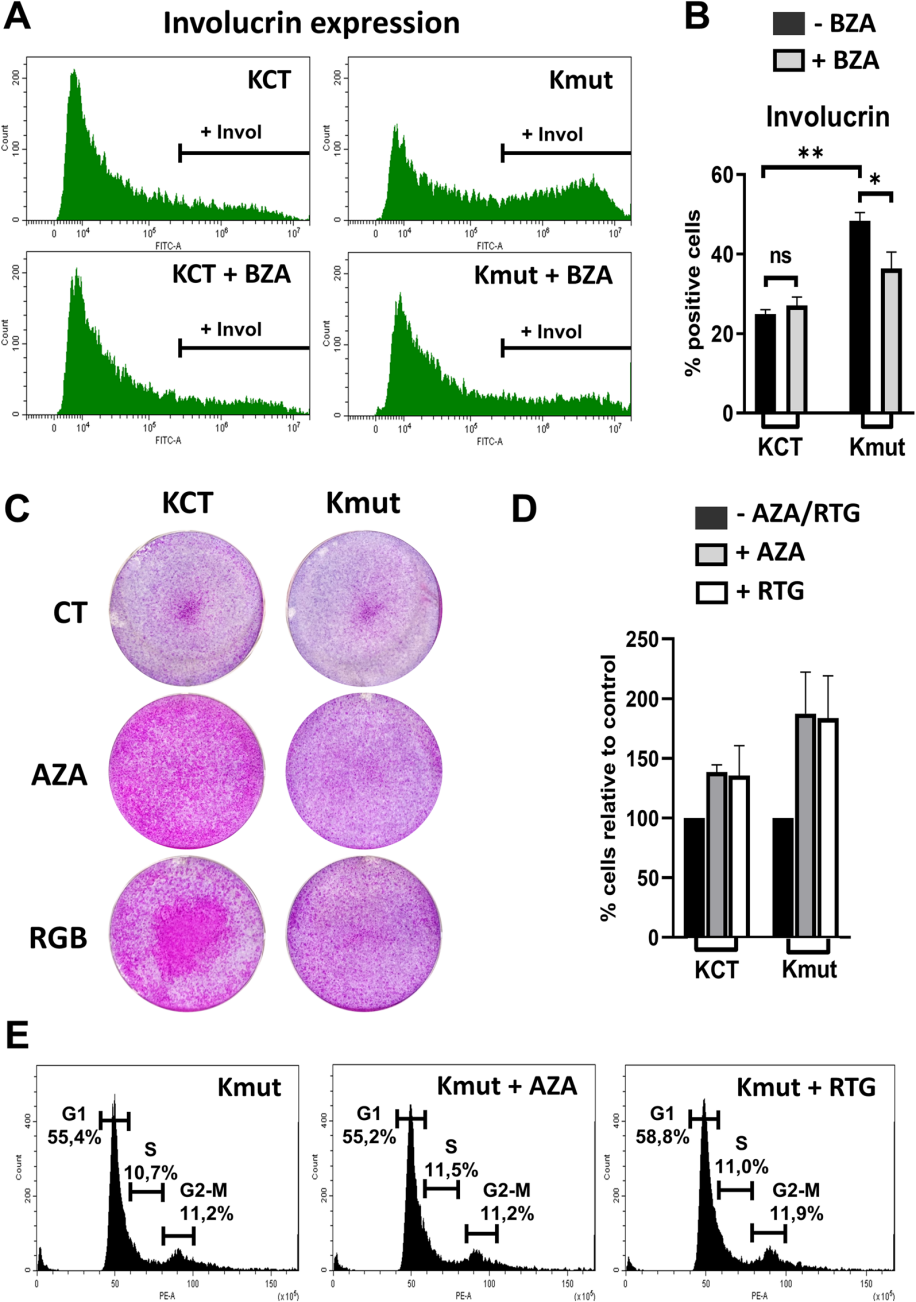
Pharmacological treatments with CAIs recover expansion capacity of the mutant primary cells isolated from the patient without significantly altered the cell cycle. **A** Representative flow cytometry histograms for the epidermoid differentiation marker involucrin of KCT or mutant Kmut cells as indicated. Cells were treated with 10 μM brinzolamide (BZA) for 6 days as indicated. **B** Bar histogram shows percentage of KCT or Kmut involucrin positive cells, as quantified by flow cytometry (*n* = 2). *ns*: non-significant. **C** Clonal expansion capacity of KCT upon 10 μM AZA or RGB 7 days treatments. Cells plated at high density, Kmut double number of cells than KCT, colonies in pink (n = 3 in each essay, four essays, with KCT cells from two different individuals). **D** Number of cells in **C**, harvested at confluence of KCT. **E** cell cycle analyses of cells in **D**, as determined by DNA content stained with Propidium Iodide. *p < 0.05, **p < 0.01

Treatment for 6 days with BZA did not cause significant changes in the growth of control KCT cultures. However, BZA caused a decrease of the involucrin positive Kmut cell population (Fig. 3A, B). AZA and RTG very significantly enhanced clonal expansion (Fig. 3C). However, the difference in the number of cells after treatments was not as striking (Fig. 3D). Furthermore, analyses of DNA content revealed no differences in cell cycle dynamics between KCT and Kmut cells (Fig. 3E). Therefore, AZA and at a higher extent RTG rescued at least in part the growth defect of Kmut cells, although did not influence proliferation rates. Other cellular processes, such as cell shape, adhesion or spreading, might be affected by the Kv10.2 LOF mutation. To note, cell shape and adhesion are critical to keratinocyte growth and differentiation [38].

**Fig. 4:**
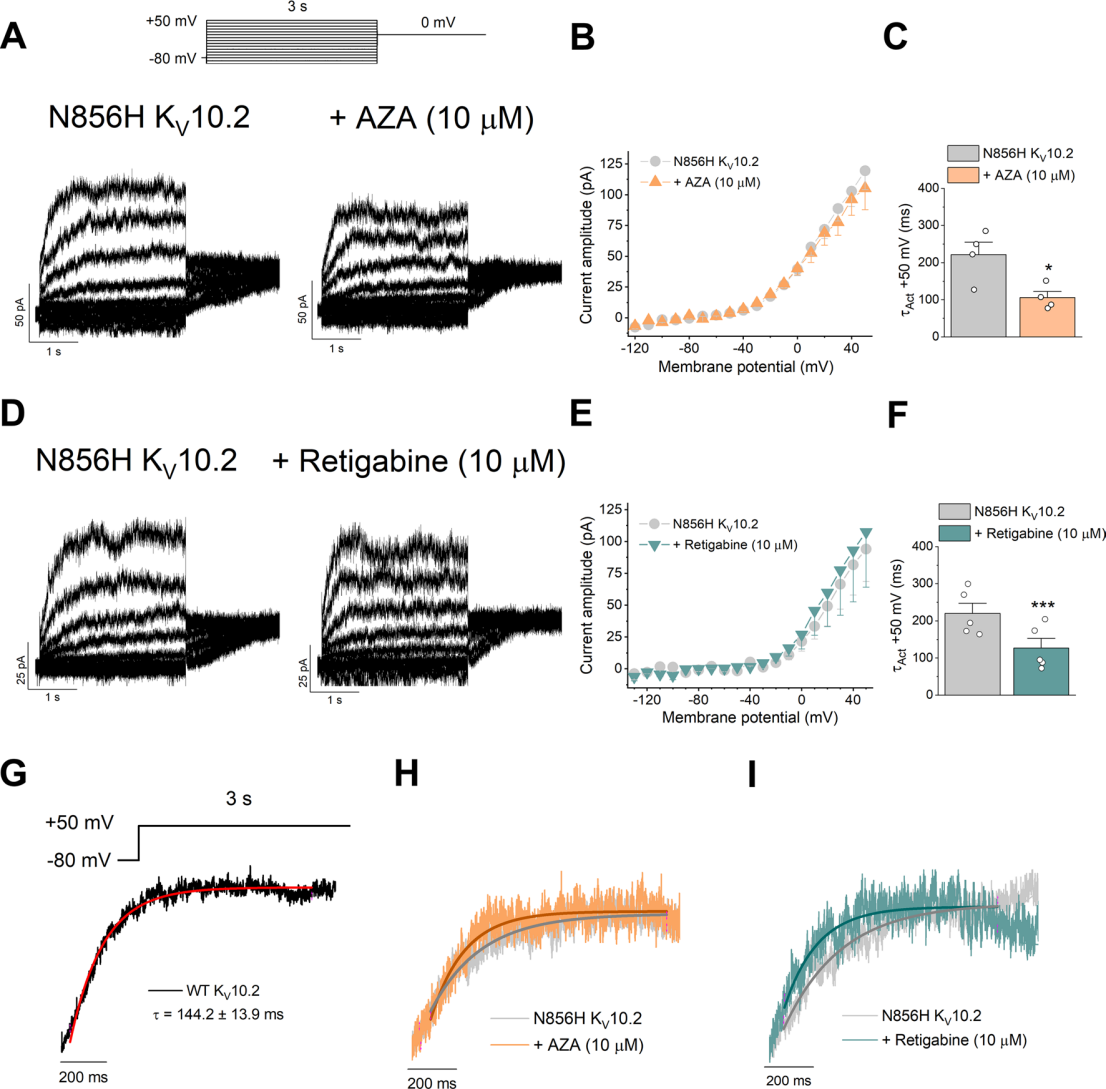
Electrophysiological measurements of N856H Kv10.2 channel expressed in COS7 cells, in presence or absence of AZA or RTG drugs. **A** Original current recordings of N856H Kv10.2 channels in the absence or presence of AZA, as indicated, after applying the pulse protocol shown on the top. Current traces are shown in 20-mV increments for clarity. **B** Current–voltage relationship obtained after plotting the amplitude of the current measured at the end of the 3-s pulses versus membrane potential in the absence and in the presence of AZA. **C** Time constant of activation of N856H Kv10.2 mutant channels at + 50 mV cells in the absence or in the presence of AZA. **D–F** As in **A–C** but with RTG instead of AZA. **G** For comparison, WT (wild type protein) Kv10.2 currents with the exponential fit of activation. **H** Current recordings elicited after the activation of N856H Kv10.2 mutant channels in the absence or presence of AZA. **I** Same as in **H** but with RTG instead of AZA. Data are expressed as the mean ± SEM of n = 4 (for AZA) and n = 5 (for RTG). *p < 0.05, ***p < 0.001

We wanted to determine whether the function of the N856H Kv10.2 was improved by the drugs described above, or the beneficial effect of treatments on Kmut cells was due to the boost of other potassium channels raising the cellular potassium influx. To that aim, we transfected again COS7 cells with the N856H Kv10.2 mutant channel protein and we performed electrophysiological analyses in the absence, or after perfusing transfected cells with AZA or RTG, at 10 μM. Figure 4 shows that N856H Kv10.2 current recordings in the absence and in the presence of AZA (Fig. 4A) or retigabine (Fig. 4D). The magnitude of the current was not modified by either drug (Fig. 4B, E). However, the time constant of activation was significantly shorter in the presence of either AZA or RTG (Fig. 4C, F). Remarkably, either AZA or RTG restored the activation kinetics of N856H Kv10.2, to levels similar of the WT Kv10.2 (Fig. 4G-I).

Finally, encouraged by the beneficial in vitro results with the CAI drugs, given that the effect of BRZ on growth was less significant (not shown) and that RTG is not authorised on minors, we introduced AZA in a combined therapeutic treatment of the patient. The aim was to improve the Kv10.2 mutated channel and to stimulate the potassium intake with AZA (250 mg every 12 h) while maintaining VPA and terbutaline treatment as described in “Materials and methods”.

To note, the clinical response to the AZA treatment by the patient was favourable with epilepsy control and significant improvement in mood (personal-social, adaptative), communication, motor and cognitive skills with no adverse effects. The treatment started at a chronological age of 10 years but a calculated neurodevelopment age of 2 to 3 year. The age-equivalent score was obtained from the development quotient (DQ) within the Battelle Developmental Inventory (BDI-2) [39]. This battery of tests analyses 5 areas of neurodevelopment: personal-social, adaptive, motor, communicative and cognitive. BDI-2 allows evaluating children from birth to a chronological age of 8 years, with some special considerations for children with developmental disorders, as in the present case [40, 41].

In 2–4 weeks of AZA combined treatment, the age equivalent representing the raw score earned by children that age, improved in all areas of neurodevelopment, being more impressive in the personal-social, communicative and cognitive skills (Table 2), as noted both by the clinician and the parents. The social, communicative, adaptive, motor and cognitive competences improved from a global DQ of average 2.3 years old (pointed out objects, interacted with the environment, poor social interaction and poor speech until complete loss) to an age-equivalent score of average 5.5 years (recovery of speech, recognised letters and completed interactive games and puzzles). The speech, which had been lost for more than 3 years, improved to 1–3 words phrases and a broad vocabulary. The social behaviour improved from a frequent apathy to being more affectionate with the family and a slight interaction with other children. As for motor skills, the patient improved from reduced walking ability to stair climbing with railing without external help. These improvements partially diminished after two months possibly due to a tolerance effect. However, 6 months after starting the treatment, in order to reduce medication, AZA intake was suppressed and 2 weeks later, the neurodevelopment significantly worsened again with epileptic seizures frequency increased. Then the treatment was re-introduced up to date.

**Table 2:** Development quotient quantification initially and after AZA treatment, according to the Battelle Developmental Inventory (BDI‐2), years of cognitive age

Gobal domains	Area assessed	Initical score	Score after treatment
Adaptative	Self‐care	2	4
	Personal responsibility	2	4
	Adult interaction	3	6
Personal‐social	Peer interaction	2	4
	Self‐concept & social role	2	6
	Receptive	3	6
Communication	Expressive	2	6
	Gross motor	2	6
Motor	Fine motor	3	5
	Perceptual motor	2	6
	Attention & memory	2	6
Cognitive	Reasoning & academic skills	2	6
	Perception & concepts	3	6
Total average improvement		2.3	5.5

## Discussion

Here we present functional, cellular and clinical evidence for the involvement of a LOF mutation of the Kv10.2 channel in a complex syndrome including mild to severe development delay, epilepsy, language and motor regression and progressive autistic behaviour. The genetic characterisation revealed a non-previously described mutation in *KCNH5* gene. This is, to our knowledge, the first LOF mutation reported in human Kv10.2 with clinical consequences. The involvement of this ion channel in epilepsy is further supported by a previously identified GOF mutation in Kv10.2 [16]. It is not striking that either loss or gain of function in a protein channel leads to a dysfunction of the channel, as the activation dynamics are anyhow affected. Recently, Kv10.2 deletion was shown to increase epilepsy susceptibility in a rat model, the authors proposing a role for Kv10.2 in the development of epileptic disorders [42]. Our results also further support suggestions that the chromosomal region around 14q23.2, where *KCNH5* is localised, might be a 'hot spot' for neurological diseases. Various clinical features associated with alterations in this chromosomal region have been described, including craniofacial abnormalities, mental retardation and epilepsy [43–45].

What caught our interest in the patient with the Kv10.2 mutation was the autistic regression with progressive language loss and the differential response to antiepileptic treatments with the same active principle (VPA) but different drug vehicles. This fact made that after discarding structural or metabolic diseases, the patient was subjected to a genetic study. Punctual mutations or hemizygous deletion of one or more critical gene(s) controlling neuronal excitability have been associated with the epilepsy phenotype [46]. Because ion channels are important determinants of seizure susceptibility and the Eag‐related voltage‐gated potassium channel Kv10.2 has been previously linked with epileptic disorders, we hypothesised that mutation of this gene may be responsible for the epilepsy phenotype.

An electrophysiological characterisation of mutated N856H Kv10.2 is the inception to elucidate its role in neurological disorder development. Even though the steady-state current amplitude was not altered in the mutant channels, their slower activation kinetics induced a significantly decreased current at short times, what would translate in a LOF of the channels during the neuron action potentials. This decrease of the current magnitude is more relevant at increased firing rates. Since the mutant channel activates more slowly, there should be less potassium efflux, promoting depolarisation of the neurons and likely, epileptic seizures. Just a few milliseconds delay in a channel opening might be enough to change the duration and rate of action potential firing, consequently disturbing the neural balance state thus leading to neurodevelopmental disorders such as epilepsy. The fact that the activation speed was restored by the AZA and RTG drugs further demonstrates that the p.Asn856His mutation causes LOF of the channel.

Interestingly, the Kv10.2 channel is widely expressed in the brain [47, 48]. Frequently, pathogenic potassium channel alterations give rise to a LOF, with reduced activity resulting in changes in the resting membrane potential and the repolarisation process [49]. As the Kv10.2 channel is expressed in pyramidal and excitatory neurons, we suggest that the mutation now identified can enhance their firing frequency. These findings would explain why restoring the potassium intake was an effective therapeutic approach for the patient.

In vitro primary skin keratinocytes characterisation showed a striking growth defect in the mutant cells freshly isolated from the patient. Keratinocyte post-mitotic differentiation is induced by various intracellular pathways, responding to growth factors, loss of cell adhesion, DNA damage or mitotic defects [50, 51]. We therefore hypothesise that the LOF mutation in Kv10.2 might indirectly disrupt some of these cellular processes. Interestingly, although the growth of mutant cells was significantly improved by the CAIs AZA and RTG, the proliferation rate (cell cycle) was not proportionally increased. Therefore, we are tempted to speculate that the LOF of the Kv10.2 channel might affect cell shape, spreading and maybe adhesion capacity. This would explain why mutant keratinocytes detached and stratified prematurely. Changes in cell shape are critical for keratinocyte homeostasis and for the axons of neurons that make synaptic connexions. Interestingly, a crosstalk between ion channel activity and the cytoskeleton has been reported and a physiological role in mechano-signalling proposed [52, 53]. Therefore, a speculative model can be drawn whereby genetic alterations might cause cognitive and epileptic disorders due to their effect on the cytoskeleton, cell shape and spreading, ultimately affecting neuron communication.

The present study not only shows a novel mutation involved in a human rare disease. It also demonstrates the utility of primary cell cultures in personalised medicine. Primary in vitro cell models can emerge as powerful systems to identify genetic mutations, cellular defects and pharmacological treatments in rare (and other) diseases. Skin or oral cell types can be isolated, expand and frozen at significant rates, without the need of repetitive biopsies [54]. As described in Results, the in vitro study due to the use of the BZA substitute equivalent in this case prompted us to modify the clinical treatment and to design a therapeutic combined strategy. The result was a remarkable improvement of the patient in oral and emotional communication, motor skills and epilepsy control. The fact that stoppage of the AZA treatment led to a behaviour regression, further points out the role of the compound in the disease control. Therefore, a general potassium intake strategy elicited good results. In order to optimise this in a more specific treatment and in view of the in vitro results, we are now considering more specific treatments by drugs such as RTG that specifically open another closely related family of voltage-gated potassium channels, Kv7.2–5 [34–37]. This might attenuate the tolerance effect observed with AZA.

The rescue of orphan drugs is an interesting strategy that may lead to finding new applications for disused compounds. An example of this is the use of CAIs in epilepsy while they were initially designed for glaucoma [28, 29, 32, 33]. However, when as in this case, personalised medicine can be implemented, a customised treatment can be designed and in vitro results can put forward the new use. Nowadays, compounds such as RTG have not been tested for its use in minors. One of the key applications of in vitro personalised studies is to validate or not the efficiency of still poorly characterised pharmaceuticals on the actual cells from the patient. These models should in the future provide significant advance in the diagnosis and treatment of rare diseases.

## Conclusions

By in vitro studying primary cells from the mutant Kv10.2 patient, we could find a readout for the cellular defects and second, test a pharmaceutical treatment that proved to be beneficial. The results show the involvement of a novel LOF mutation of a Potassium channel in an epilepsy/autism syndrome and provide potential new understanding into neurological genetic rare diseases. In summary, our study proves the great potential of in vitro cultures of primary cells in personalised medicine of rare (and other) diseases that should be further exploited.

## Materials and methods

### Clinical treatments

The patient received various antiepileptic treatments with variable impact not only in epilepsy control but also in the neurodevelopment and autistic behaviour. Treatment with valproate (400 mg every 12 h), drug enhancing GABAergic transmission, was associated with good epilepsy control and acquisition of new skills. Treatment with terbutaline (3 mg every 12 h) was applied to improve potassium transport via the sodium–potassium pump β-adrenergic-mediated [55–58]. Otherwise, we tested and suppressed treatment with levetiracetam, antiepileptic that inhibits the synaptic vesicle 2A, because of sudden epileptic seizures followed by periods of stagnation or regression in neurodevelopment.

### Exome sequencing, processing and analysis

First, we performed an exome sequencing for > 1,870 genes associated with neuropediatric diseases using a Neuroexome gene panel provided by CGPP-IBMC (Spain), followed by a Trio Exome sequencing, with samples of the patient and both parents. For the Neuroexome panel, the next-generation sequencing (NGS) library was prepared using a customized SureSelect XT kit from Agilent (ref. S3133972, Santa Clara, CA, USA) followed by paired-end sequencing on a NexSeq500 from Illumina (San Diego, CA, USA). For the Trio Exome, the NGS library was prepared using a SureSelect Human All Exon V6 kit from Agilent (ref. S07604514), which capture 60 MB of the human exome target sequence, subsequently sequenced on a Novaseq 6000 from Illumina.

For the Neuroexomegene panel, the average exome sequence coverage per nucleotide was 353X, a 98.36% of the target exome sequences captured a minimum of 20 × reads, and a 99.22% were higher to 10x. For the Trio Exome, the average exome sequence coverage per nucleotide was 292X, a 97.4% of the sequences captured a minimum of 20x, and a 97.9% were higher to 10x. To search possible deletions/insertions a personalised Software was used, PattRec [59].

Allele frequencies of the previously known variants were annotated according to gnomAD and TOPMED databases. We displayed GERP score to measure the conservation of the mutated base [60], and CADD tool to measure the predicted deleteriousness of the nucleotide variants [61]. To predict the pathogenicity of missense variants we used the REVEL method, which integrates scores from 13 different prediction tools [62, 63]. Finally, we used ClinVar database to search relationships between genetic variations reported for a gene and associated patient phenotypes [64].

### Plasmids and site-directed mutagenesis

The human Kv10.2 cDNA cloned in pcDNA3.1 vector was kindly provided by Luis A. Pardo (Max Planck Institute for Multidisciplinary Sciences, Göttingen, Germany). Point mutation in wild-type Kv10.2 was introduced by PCR, using Herculase II Fusion DNA polymerase (Agilent, Santa Clara, CA USA). PCR cycle programs were as follows: initial denaturation for 4 min at 96 °C, followed by 30 cycles consisting of: 30 s at 96 °C, 1 min at 55 °C and 8 min at 72 °C. After these cycles, another period of 30 s at 72 °C. After these cycles, another period of 30 s at 72 °C. Reference nucleotide sequence used was NG_034062. The patient exhibited a heterozygous potentially pathogenic missense mutation (c.2566A > C; p.Asn856His) affecting a potassium channel (Kv10.2). To that end, the following primers were used (modified sequence is shown in underlined letters):

Forward: 5′ CCAAA**C**ACCCACTAAGAAAAACAG 3′

Reverse: 5′ GTGGGTGTTTGGT**C**ACACTG 3′

The product of the PCR was purified with QIAquick Gel Extraction Kit (Qiagen, Germany) and then digested with *DpnI* (New England Biolab, Ipswich, MA, USA) for 1 h at 37 °C and 15 min at 70 °C in order to remove the traces of the original vector and obtain only the DNA resultant of the PCR. Wild-type and mutant constructs were verified by DNA sequencing.

### Cell culture and transient transfection

Primary keratinocytes were isolated and cultured in the presence of a mouse fibroblast feeder layer (inactivated by mitomycin C), in Rheinwald FAD medium supplemented with 10% (v/v) foetal bovine serum (FBS; Gibco, Paisley, UK), 1.2 mM Ca^2+^, 5 ng/ml Epidermal growth factor (EGF) and 100 U/ml of penicillin/streptomycin (P/S, Gibco), as described previously [65, 66]. Low passages (2–4) keratinocytes from the patient and two independent healthy individuals were included in the studies. Mouse fibroblast 3T3-J2 cell line used as feeder layer was cultured in Dulbecco’s Modified Eagle’s Medium (DMEM, Gibco) supplemented with 10% (v/v) of donor calf serum and 100 U/ml of P/S. Brinzolamide solution (AZOPT, Novartis, Switzerland), Acetazolamide (AZA, ref. A6011, Sigma-Aldrich) and retigabine (RTG, ref. 90,221, Supelco) were used in cultures at 10 μM.

For electrophysiology experiments, COS7 cells were cultured in DMEM supplemented with 10% (v/v) of FBS and 100 U/ml of P/S and plated in 35-mm culture dishes. Cells were transiently cotransfected with WT Kv10.2 or N856H Kv10.2 cloned into pCDNA3.1, and EBO-pcDLeu2 as reporter gene, codifying CD8. This was performed by using Fugene-6 (Merck, Germany) at 60% confluence and following manufacturer’s instructions [67, 68]. The amount of cDNA transfected was 4 μg for the channel and 2 μg for EBO and the ratio of DNA:Fugene was 1:3. After 48 h transfection, cells were removed from culture plates using TrypLE™ Express (Gibco), after exposing cells to polystyrene microspheres bound to anti-CD8 (Dynabeads M-450; Thermo Fisher Scientific, Waltham, MA, USA) [67–69].

### Electrophysiology

Potassium currents were recorded from COS7 cells using the whole-cell configuration of the patch-clamp technique with an Axopatch 200B amplifier and a Digidata 1322A (Molecular Devices, Silicon Valley, CA, USA) as previously described [70–72]. The intracellular pipette filling solution contained: 80 mM K-aspartate, 50 mM KCl, 3 mM phosphocreatine, 10 mM KH_2_PO_4_, 3 mM MgATP, 5 mM HEPES-K, 5 mM EGTA and was adjusted to pH 7.25 with KOH. The bath solution contained: 145 mM NaCl, 4 mM KCl, 1.8 mM CaCl_2_, 1 mM MgCl_2_, 10 mM HEPES-Na, 10 mM glucose and was adjusted to pH 7.40 by NaOH.

Currents were filtered at 1 kHz (4-pole Bessel filter) and sampled at 2 kHz. Micropipettes were pulled from borosilicate glass capillary tubes (GD-1; Narishige, Japan) on a programmable horizontal puller P-87 (Sutter Instruments Co., Novato, CA, USA) and heat-polished with a microforge (Narishige). Micropipette resistance ranged between 2–4 MΩ. pClamp version 10.6 software (Molecular Devices) was used for data acquisition and analysis. Currents were recorded at room temperature (21–23 °C) at a stimulation frequency of 0.03 Hz. OriginPro 2018 (OriginLab Corporation, Northampton, MA, USA.) and Clampfit 10.6 programs were used to perform least-squares fitting and for data presentation. All the compounds used for the external and internal solutions were from Merck.

### Clonogenicity assays

For clonogenicity assays, 5,000 keratinocytes per well were plated per triplicate in 6-well plates and grown in high-calcium FAD medium. About 14 days later, the cultures were fixed with 3.7% formaldehyde in PBS for 10 min and stained with rhodanile blue as described previously [25].

### Flow cytometry and Antibodies

Keratinocytes were harvested, fixed in 3.7% formaldehyde in PBS and stained for involucrin as previously described [38]. Primary anti-involucrin antibody (SY5, I-9018, Sigma-Aldrich, Inc., St Louis, MO, USA) produced in mouse, then revealed with Alexa Fluor® 488-conjugated goat anti-mouse (115-547-003) from Jackson ImmunoResearch (Philadelphia, PA, USA). Antibody staining was controlled by the use of a similar concentration of isotype negative immunoglobulin IgG from mouse serum (I-5381, Sigma-Aldrich). After staining, cells were firmly resuspended and filtered through a 70 µM mesh to minimize the presence of aggregates and then analysed on a CytoFLEX from Beckman Coulter (Brea, CA, USA). 10,000 events were gated and acquired to analyse.

### Statistical analyses

Data are presented as mean ± standard deviation from two or more independent culture dishes conditions (*n*) and at least two independent experiments (*N*). Data sets were compared using an unpaired two-tailed Student’s t test when two data sets or one-way ANOVA when more than two data sets were analysed (GraphPad Prism 8). For multiple comparison, Tukey test was used. A *p* value of < 0.05 was considered statistically significant. In every case sample size was chosen accordingly. Damaged samples were excluded from analyses.

## Data Availability

All data generated or analysed during this study are included in this published article.

## Abbreviations

ASD: Autism spectrum disorder
AZA: Acetazolamide
BZA: Brinzolamide
CAI: Carbonic anhydrase inhibitor
DMEM: Dulbecco’s Modified Eagle’s Medium
Eag: Ether-à-go-go
EGF: Epidermal growth factor
ES: Exome sequencing
FBS: Fetal bovine serum
FDA: Food and Drug Administration
GOF: Gain of function
HS: High scatter
LOF: Loss of function
MRI: Magnetic Resonance Imaging
NGS: Next-generation sequencing
P/S: Penicillin/streptomycin
RTG: Retigabine
VPA: Valproate
WT: Wildtype
